# Hypolipidemic and Anti-Atherogenic Effects of Sesamol and Possible Mechanisms of Action: A Comprehensive Review

**DOI:** 10.3390/molecules28083567

**Published:** 2023-04-19

**Authors:** Amin F. Majdalawieh, Aaram E. Eltayeb, Imad A. Abu-Yousef, Sarah M. Yousef

**Affiliations:** Department of Biology, Chemistry, and Environmental Sciences, College of Arts and Sciences, American University of Sharjah, Sharjah P.O. Box 26666, United Arab Emirates; g00086929@aus.edu (A.E.E.);

**Keywords:** sesamol, *Sesamum indicum*, macrophages, cholesterol, lipids, atherosclerosis

## Abstract

Sesamol is a phenolic lignan isolated from *Sesamum indicum* seeds and sesame oil. Numerous studies have reported that sesamol exhibits lipid-lowering and anti-atherogenic properties. The lipid-lowering effects of sesamol are evidenced by its effects on serum lipid levels, which have been attributed to its potential for significantly influencing molecular processes involved in fatty acid synthesis and oxidation as well as cholesterol metabolism. In this review, we present a comprehensive summary of the reported hypolipidemic effects of sesamol, observed in several *in vivo* and *in vitro* studies. The effects of sesamol on serum lipid profiles are thoroughly addressed and evaluated. Studies highlighting the ability of sesamol to inhibit fatty acid synthesis, stimulate fatty acid oxidation, enhance cholesterol metabolism, and modulate macrophage cholesterol efflux are outlined. Additionally, the possible molecular pathways underlying the cholesterol-lowering effects of sesamol are presented. Findings reveal that the anti-hyperlipidemic effects of sesamol are achieved, at least in part, by targeting liver X receptor *α* (*LXRα*), sterol regulatory element binding protein-1 (*SREBP-1*), and fatty acid synthase (*FAS*) expression, as well as peroxisome proliferator-activated receptor α (*PPARα*) and AMP activated protein kinase (*AMPK*) signaling pathways. A detailed understanding of the molecular mechanisms underlying the anti-hyperlipidemic potential of sesamol is necessary to assess the possibility of utilizing sesamol as an alternative natural therapeutic agent with potent hypolipidemic and anti-atherogenic properties. Research into the optimal sesamol dosage that may bring about such favorable hypolipidemic effects should be further investigated, most importantly in humans, to ensure maximal therapeutic benefit.

## 1. Introduction

Hyperlipidemia is one of the most prevalent risk factors for the development of atherosclerosis and consequent cardiovascular diseases (CVDs) [1]. It is mainly characterized by significantly high levels of serum total cholesterol (TC), triglyceride (TG), and low-density lipoprotein cholesterol (LDL-C) levels [2]. Previous studies consistently showed that elevated levels of LDL-C contribute substantially to the formation of atherosclerotic plaques, highlighting the critical role that this lipid plays in CVD development [1]. Drugs that aim to lower LDL-C levels are therefore commonly used to help mitigate or treat CVDs [3]. Out of these drugs, statins have gained considerable popularity and have been extensively developed and employed over the years to treat hyperlipidemia in humans [4]. Although effective, the use of statins, particularly those that are of synthetic origin, to treat hyperlipidemia may pose problems due to the inevitable adverse effects associated with their usage [5]. Recently, there has been a rise in interest in employing natural alternatives to treat hypercholesterolemia. A class of substances known as lignans is of particular importance in this context. Lignans are phenolic dimers derived from cis-o-hydroxycinnamic acid and are found copiously in plants such as flaxseeds, sesame seeds, and brassica vegetables [6,7]. In particular, sesame seeds are one of the most prevalent plant sources high in these lignans and, as such, have drawn a great deal of attention owing to their abundant cultivation in Africa and Asia [8] and their potent anti-atherogenic properties [9].

Sesame (*Sesamum indicum* L.) belongs to the Pedaliaceae family and is composed of lignans such as sesamin, sesamol, sesaminol, and sesamolin, with only sesamol being an industrially synthesized lignan [10,11]. The therapeutic potential of these lignans was investigated in several *in vivo* and *in vitro* studies, and there is strong evidence indicating that these lignans provide a number of physiological and medicinal benefits [12]. Sesamol, 5-hydroxy-1,3-benzodioxole, or 3,4-methylene-dioxyphenol, is a water-soluble phenolic lignan found in sesame seeds and sesame oil [13]. The molecular formula of sesamol is C_7_H_6_O_3_, its molar mass is 138.12 g/mol, and it can be found in trace amounts in sesame oil [14]. Even though sesamol has been reported to possess beneficial pharmacological properties, it is not as widely researched as the other lignans found in *S. indicum*. This bioactive compound was demonstrated to exert potent antioxidant [15,16], anti-inflammatory [17,18,19], chemo preventive [20], anti-cancer [21], cardioprotective [22], anti-atherogenic [23,24,25] and, most relevant to this review, cholesterol-lowering and hypolipidemic effects. Despite this evidence, no reviews have been conducted on the underlying mechanisms of the hypolipidemic and anti-atherogenic effects of sesamol. This review aims to shed light on these effects by highlighting the main hypolipidemic and anti-atherogenic properties of sesamol emphasizing its beneficial role in managing lipid profiles. Additionally, major potential mechanisms of action and pathways underlying the antihyperlipidemic effects of sesamol will be underscored by reflecting on the role this lignan has on fatty acid oxidation, synthesis, cholesterol metabolism, and macrophage cholesterol efflux. Understanding such mechanisms of action is crucial, as they offer a solid scientific basis to support the anti-hyperlipidemic properties of sesamol and its role in improving cardiovascular health.

## 2. Search Methodology

The literature search was conducted using a variety of online databases, such as PubMed, Cochrane Library, Elsevier (Science Direct), and Google Scholar. To ensure the identification of relevant studies, the word “sesamol” was used along with keywords including, but not limited to, “fatty acid synthesis”, “fatty acid oxidation”, “cholesterol metabolism”, “macrophage”, “lipid”, and “atherosclerosis”. Articles were screened and selected with no restriction on publication dates, the experimental subjects/models used, the mode and duration of sesamol administration, or other experimental details.

## 3. Effects of Sesamol on Lipid Profile

Cholesterol and TGs are lipophilic lipid molecules that are transported within the plasma in combination with molecules known as lipoproteins, including LDL-C, very low-density lipoprotein (VLDL), and high-density lipoprotein cholesterol (HDL-C) [26]. Abnormal levels of lipoproteins are known to increase the likelihood of developing CVDs, whereby elevated levels of LDL-C lead to an accumulation of atherosclerotic plaques within the arteries [27]. HDL-C mediates molecular pathways that eliminate cholesterol from the body, and therefore elevated levels of HDL-C have been shown to lower the risk of CVDs [28]. Given the significant effects a hypolipidemic agent can have on the prevention and management of CVDs, several *in vivo* studies were conducted to assess the hypolipidemic role that sesamol can play in various animal models. In one *in vivo* study, Sharma and colleagues assessed the molecular mechanisms of sesamol for preventing high-fat diet (HFD)-induced cardiometabolic syndrome (CMetS) in male Wistar albino rats, including its effect on hyperlipidemia [29]. Notably, oral administration of 2, 4, and 8 mg/kg/day of sesamol for 30 days was shown to inhibit HFD-induced hyperlipidemia in rats, whereby sesamol exerted a dose-dependent attenuation of elevated TC, TG, and LDL-C levels along with a concomitant elevation in HDL-C concentration [29]. Further analysis from the same study revealed that the sesamol-mediated attenuation of hyperlipidemia was found to be mediated, at least in part, via upregulating hepatic peroxisome proliferator-activated receptor α (*PPARα*) expression and downregulating the expression of liver X receptor α (*LXRα*), sterol regulatory element binding protein 1c (*SREBP-1c*), and fatty acid synthase (*FAS*), indicating reduced lipid content [29]. These findings were further corroborated in another *in vivo* study that examined the therapeutic effects of sesamol on obesity induced by a HFD in male C57BL/6J mice [30]. Specifically, Qin and colleagues found that administering 100 mg/kg body weight/day of sesamol to the mice markedly promoted lipid metabolism and exerted significant hypercholesterolemic effects via reducing serum TC and LDL-C levels and elevating HDL-C levels [30]. Although promising, research looking at the pathways involved in producing such hypolipidemic effects is warranted to reaffirm the role of *PPARα*, *LXRα*, *SREBP-1c*, and *FAS* in reducing lipid levels. Additionally, it would be interesting to examine the effects of sesamol on apolipoprotein A (ApoA) and apolipoprotein B (ApoB), the main structural proteins found in LDL and HDL, respectively, to gain a deeper understanding of the molecular mechanisms mediating the effects of sesamol on these lipids. Similarly, in another *in vivo* study on HFD-induced obese male C57BL/6J mice, sesamol administration (100 mg/kg body weight/day) for 8 days was found to suppress lipid accumulation and cause a significant reduction in both TC and TG levels [31]. In another *in vivo* study on the effects of sesamol on aluminum chloride-induced changes in male Wistar rats, sesamol was shown to reduce dyslipidemia by reversing the changes in serum lipids caused by aluminum chloride treatment [32]. Specifically, pretreating Wistar rats orally with 10 and 20 mg/kg of sesamol 45 min prior to the administration of aluminum chloride for 60 days yielded significant antihyperlipidemic results compared to the non-sesamol-treated group, in which the levels of TG, TC, and LDL-C were significantly reduced [32]. In addition, the level of HDL, which had been reduced by lipid metabolism modifications caused by the hepatic accumulation of aluminum, was elevated in the presence of sesamol, suggesting enhanced cholesterol-scavenging atheroprotective potency [32]. In another *in vivo* study, sesamol was found to reduce the levels of TG and TC in three models of hyperlipidemia in male Swiss Albino mice [33]. In the first part of this study, a model of olive oil-induced acute hypertriglyceridemia was treated with 100 and 200 mg/kg of sesamol to assess the sesamol-mediated effects on the lipid absorption pathway [33]. Sesamol was found to reduce the elevated TG levels in a concentration-dependent manner, indicating that the hypolipidemic potential of sesamol is comparable to that of orlistat, a drug used to treat obesity [33]. Moreover, a tyloxapol-induced model of hyperlipidemia was treated with 200 mg/kg of sesamol to evaluate changes in lipid metabolism [33]. The results obtained from this model revealed that sesamol reversed the elevated TG levels, normalized the TC level, and counteracted the tyloxapol-induced elevation of plasma cholesterol [33]. In addition, sesamol (50 and 100 mg/kg) was shown to reduce both TC and elevated TG levels when administered to a model of chronic hyperlipidemia in mice induced by a high-fat and high-fructose diet (HFFD), which closely resembles the hyperlipidemia condition in humans [33]. In another *in vivo* study, the protective effects of sesamol against atherosclerosis were evaluated in HFD-induced male Syrian hamsters [34]. Sesamol (50 or 100 mg/kg) ingestion by oral gavage for 16 weeks was shown to exert lipid-lowering effects on elevated serum lipid levels, which would typically predispose an individual to atherosclerosis, whereby sesamol reduced the serum levels of TC, LDL-C, and VLDL, a triglyceride-rich lipoprotein that transports TGs synthesized in the liver [34,35]. Additionally, sesamol (50 or 100 mg/kg) dose-dependently reduced the plasma level of L5, a highly electronegative type of LDL that has been shown to be at elevated levels in people with high risks for CVDs such as hypercholesterolemia [34]. Moreover, the measured size of atherosclerotic lesions in the aortas of the Syrian hamsters was found to be significantly reduced in the presence of sesamol, indicating compelling evidence to support the hypolipidemic potential of sesamol [34]. In a similar *in vivo* study, sesamol has been shown to attenuate changes in lipid metabolism in isoproterenol (ISO)-induced male albino Wistar rats via its cardioprotective and antihyperlipidemic properties [36]. In particular, Vennila and colleagues reported that the ISO-induced myocardial infarction in the rats resulted in elevated plasma TC, LDL-C, and VLDL levels, whereas it also reduced the plasma HDL-C level. Given that LDL-C deposits cholesterol in the arterial walls, which contributes to atherosclerosis, while HDL-C has anti-atherogenic properties, these ISO-induced changes in plasma lipids increase susceptibility to CVDs [36]. Notably, the intraperitoneal administration of 50, 100, and 200 mg/kg of body weight of sesamol for 7 days resulted in the return of plasma TC, LDL-C, HDL-C, and VLDL to near-normal levels [36]. Furthermore, the elevated levels of plasma TGs were also reduced in the presence of sesamol, and the results of this study indicated that the optimum concentration of sesamol for exerting hypolipidemic effects is 50 mg/kg of body weight [36]. In another *in vivo* study that investigated the protective effects of sesamol against doxorubicin (DXO)-induced cardiomyopathy in Wistar rats, sesamol was shown to prevent myocardial necrosis through significant lipid-lowering effects [37]. Pretreating Wistar rats with 50 mg/kg of body weight of sesamol for 7 days followed by DXO administration for 2 weeks was found to markedly reduce (*p* < 0.05) serum TC, TG, LDL-C, and VLDL levels and simultaneously elevate (*p* < 0.05) HDL-C levels [37]. The lipid-lowering effects reported in this study and others suggest that sesamol may be employed as a potential therapeutic agent for treating CVDs.

Sesamol was also shown to exhibit its lipid-lowering effects on intracellular lipid levels. For instance, in a combined *in vivo* and *in vitro* study, Xu and colleagues explored the effects of sesamol on the treatment of hepatic steatosis in HFD-induced obese mice and palmitic acid (PA)-induced HepG2 cells [38]. Notably, and similar to the findings on hepatic liver profiles that were previously reported by Qin and colleagues [30], the ingestion of 100 mg/kg body weight/day of sesamol by gavage significantly improved hepatic liver profiles *in vivo*, whereby sesamol reduced hepatic fat vacuoles and liver weight, in addition to reducing serum ALT and AST levels as well as hepatic TG and LDL-C levels [38]. Such sesamol-mediated effects on the hepatic liver profile indicate the improvement of liver function and the alleviation of hepatic steatosis [38]. In another *in vitro* investigation, high oleic acid/cholesterol-induced HepG2 cells were treated with sesamol to study its protective effects against hepatic steatosis [39]. Sesamol (0.75, 1.5, and 3 μg/mL) was shown to significantly reduce the intracellular TG and TC levels by activating the peroxisome proliferator-activated receptor alpha (*PPAR*) signaling pathway [39]. Further analysis revealed a reduction in lipid droplets (LDs) in the presence of sesamol (1.5 and 3 μg/mL) which suggests both the reduction in fatty acid synthesis and the elevation of lipolysis [39].

In addition to these reported findings on sesamol, a number of sesamol derivatives were also found to demonstrate potent anti-hyperlipidemic properties. Interestingly, in an *in vivo* investigation, Xie and colleagues synthesized a series of 12 sesamol-based fibrate derivatives (numbered 1 to 12) to observe their hypolipidemic effects on Triton WR-1339-induced hyperlipidemic mice [40]. Results from this study revealed that all the derivative compounds caused a reduction in the hyperlipidemic activity of the mice, with compound **12** shown to exert the most significant lipid-lowering effects on serum lipid levels [40]. In particular, compound **12** was shown to cause a dose-dependent reduction in serum TG and TC levels, with 1.086 mmol/kg body weight of compound **12** causing a 74.1% reduction in TG levels and a 70.1% reduction in TC levels [40]. Similarly, compound **12** was also shown to significantly reduce LDL-C levels in HFD-induced hyperlipidemic mice [40]. Further molecular investigation of this compound also revealed that the reported lipid-lowering effects of compound **12** were mediated, at least in part, via activating the PPARα pathway [40]. In another *in vivo* investigation by the same research group, the hypolipidemic effects of newly synthesized sesamol-based phenolic acid derivative compounds were assessed in two models of hyperlipidemia [41]. In the preliminary study of the fifteen derivative compounds, 0.36 mmol/kg of compounds **T1**–**T15** were intragastrically administered to a model of acute hyperlipidemia induced by Triton WR 1339 in Kunming (KM) mice for 7 days [41]. Although most of the derivative compounds were found to reduce plasma TG and TC levels, compound **T6** (benzo[d][1,3]dioxol-5-yl 3,4,5-trihydroxybenzoate) was shown to exhibit the strongest lipid-lowering effect on the hyperlipidemic mice, whereby T6 reduced the plasma TG level by 79.01% and the plasma TC level by 69.02%, as compared to the control group [41]. To further investigate the chronic hypolipidemic effects of compound **T6**, the same dosage (0.36 mmol/kg) was intragastrically administered to HFD-induced hyperlipidemic KM mice for 4 weeks, and **T6** was shown to exert significant lipid-lowering effects on the mice [41]. Further molecular analysis revealed that compound **T6** reduced the plasma levels of TG, TC, and LDL-C by activating the PPARα receptor, which suggests that T6 could function as a potent hypolipidemic agent [41]. Although promising, further *in vivo* and *in vitro* studies are certainly warranted to better understand the mechanisms of this newly synthesized compound. In a similar *in vivo* study, Xie and colleagues examined the effects of sesamol-clofibrate (CF-sesamol), a compound containing sesamol and clofibric acid pharmacophores, on hyperlipidemia in Triton WR 1339-induced KM mice. Notably, the lipid-lowering effects of CF were found to be similar to those reported on compound **T6** as declared in the previous study by Xie et al. [42]. Precisely, treating mice with 0.36 mmol/kg of CF-sesamol intragastrically for 4 weeks was found to reduce plasma TG levels by 38.6% and TC levels by 35.1%, demonstrating higher hypolipidemic activity than administering clofibrate and sesamol separately [42]. The findings of this study strongly support the hypothesis that CF-sesamol can be used to treat hyperlipidemia. Although promising, research suggests that other lignans found in sesame seeds, such as sesamin, also exert potent lipid-lowering effects [43]. The question remains as to how the hypolipidemic effects of sesamol relate to those of sesamin and which lignan is believed to exert superior lipid-lowering effects. Such investigation will aid in the development of hypolipidemic drugs employing agents that exert the most effective lipid-lowering properties.

The hypolipidemic and hypocholesterolemic effects of sesamol that were observed in the aforementioned studies indicate the potential of using sesamol as a potent therapeutic treatment against hyperlipidemia, which would in turn reduce the risk for CVDs such as atherosclerosis and myocardial infarctions. The sesamol-mediated activation of *PPARα* was the primary molecular mechanism associated with the lowering of serum lipid levels in these studies. However, these *in vivo* anti-hyperlipidemic effects of sesamol were only observed in animal models, including mice, rats, and hamsters. In a functional genomic study, findings revealed discrepancies in differential gene expression between HFD-induced rats and humans, and similar results were reported by Geerts, with many functional genotypes absent in rodent models [44,45]. Hence, the issue of genetic relatedness poses the question of whether these sesamol-mediated hypolipidemic effects would also be observed in humans. Therefore, further *in vivo* and *in vitro* studies, as well as clinical trials, are certainly warranted to evaluate and establish the role that sesamol may play in lowering lipid levels. The reported *in vivo* and *in vitro* effects of sesamol and its derivatives on serum lipid profiles are summarized in Table 1.

## 4. Possible Mechanisms of Action Modulating the Hypolipidemic Role of Sesamol

### 4.1. Effects of Sesamol on Fatty Acid Synthesis

Fatty acids serve as both energy sources and membrane constituents, and they influence many biochemical processes in the human body [46]. However, substantial evidence suggests that elevated fatty acids may increase the risk of CVDs, type 2 diabetes, and even cancer [46]. Studies have also shown that replacing saturated fatty acids with unsaturated fatty acids reduces LDL-C, which in turn lowers CVD risk [47]. Although the exact mechanism has not been well established, there are numerous studies in different experimental models demonstrating that sesamol can inhibit fatty acid synthesis and reduce lipid accumulation. For example, in an *in vitro* investigation on the effects of sesamol on obesity using 3T3-L1 cells, Go and colleagues reported that treating 3T3-L1 cells with sesamol (0, 50, 100, and 150 μM) for 24 h resulted in a dose-dependent reduction in the expression levels of *FAS* and *SREBP-1*, both of which play pivotal roles in promoting fatty acid synthesis. Molecular analysis revealed that this sesamol-mediated drop in *FAS* and *SREBP-1* levels is due, at least in part, to the sesamol-mediated activation of the AMP-activated protein kinase (AMPK) signaling pathway [48]. In a similar *in vivo* study by Sharma and colleagues in HFD-induced cardiometabolic syndrome in male Wistar albino rats, sesamol (2, 4, and 8 mg/kg/day) administration was shown to downregulate *FAS* and *SREBP-1* expression, which caused a significant reduction in fatty acid synthesis [29]. These results were further corroborated in another *in vitro* investigation, whereby sesamol was found to downregulate fatty acid synthesis and elevate lipolysis in HepG2 cells [39]. Precisely, sesamol (0.75, 1.5, and 3 μg/mL) treatment for 36 h was found to reduce lipid droplets (LDs), indicating reduced fatty acid synthesis and elevated lipolysis activity [39].

Sesamol was also shown to possess fatty acid metabolizing effects through its ability to inhibit key events in fatty acid synthesis. For example, in one *in vivo* study, the effects of sesamol on the fatty acid composition of the liver membrane phospholipids were examined using female BALB/c mice that were orally fed a diet containing 5 wt% safflower oil and 1 wt% sesamol [49]. Sesamol was found to be associated with a reduction in the Δ5 desaturation index for ω−6 fatty acids, evidenced by an increased accumulation of dihomo-γ-linolenic acid (DGLA) and a reduction in arachidonic acid (AA) levels in the liver membrane phospholipids [49]. These results indicate reduced synthesis of polyunsaturated fatty acids (PUFAs), contributing to reduced fatty acid synthesis. Further investigations also revealed that the levels of docosapentaenoic acid (DPA) were noticeably lower in the presence of 1% sesamol, whereas the amounts of saturated and monounsaturated fatty acids and linoleic acid were not altered [49]. Although promising, previous lines of evidence precluded the ability of sesamol alone to inhibit the Δ5 desaturase *in vitro*, suggesting that these fatty acid synthesis-inhibiting effects are rather attributed to a sesamol metabolite [49]. Further *in vitro* and *in vivo* studies are certainly warranted to confirm this claim. In another combined study, Wynn and colleagues found that sesamol (0–10.1 mM) exerted exceptional lipid-lowering effects *in vivo* in *Mucor circinelloides*, an oleaginous species of fungus, whereby sesamol caused a significant reduction in the fatty acyl composition of the cell lipid produced [50]. Precisely, sesamol (1.5 mM) was found to considerably reduce the activity of Δ6 and Δ12 desaturase enzymes [50]. Interestingly, previous *in vivo* and *in vitro* studies conducted on other lignans found in sesame seed, mainly sesamin, were found to reduce fatty acid synthesis not via Δ6 or Δ12 desaturases but rather Δ5 desaturases. It may be useful to investigate the role that sesamol may play against Δ5 desaturase specifically to see if similar inhibiting effects are at play. Additionally, the sesamol-mediated reduction in this study was accompanied by a concomitant gradual increase in the concentrations of oleic acid (18:1) and stearic acid (18:0), which is indicative of increased saturated fatty acid and decreased unsaturated fatty acid content [50]. Interestingly, Wynn and colleagues noticed that the sesamol-mediated effects on lipid metabolism were exclusive to *M. circinelloides* and not to other microorganisms, as the addition of sesamol (7.2 mM) was not found to be accompanied with any decrease in fatty acid desaturation in the other microorganisms [50]. Molecular analysis from this study also revealed that sesamol exerted its lipid-lowering effects *in vivo* via reducing the activities of NADP+/isocitrate dehydrogenase, pyruvate carboxylase, the fatty acid synthetase complex, and malic enzyme [50]. A reduction in nicotinamide adenine dinucleotide phosphate (NADPH) supply, which is required for fatty acid synthesis and desaturation, was reported as a result of the lipid metabolism changes mediated by sesamol [50]. Surprisingly, sesamol was not found to result in the same enzyme-lowering effects *in vitro*, as further analysis revealed that the observed lipid-lowering effects in this study were not due to sesamol but rather to one of its metabolites that was exclusively produced and assimilated following sesamol metabolism in *M. circinelloides* [50]. Further studies are warranted to evaluate the *in vivo* and *in vitro* effects of sesamol on the activity of Δ5 and Δ6 desaturases. In another *in vivo* study, Liu and colleagues investigated the effects of sesamol (0, 0.5, 1, 1.5, and 2 mM) on the synthesis and productivity of docosahexaenoic acid (DHA), a polyunsaturated fatty acid, in *Crypthecodinium cohnii* [51]. Notably, the presence of 0.5 mM sesamol caused a reduction in the total fatty acid (TFA) content by 25.24%, accompanied by a concomitant decrease in reactive oxygen species (ROS) levels, suggesting that sesamol reduces the synthesis of fatty acids by reversibly inhibiting the intracellular ROS [51]. Further analysis using fluorescence intensity revealed that sesamol resulted in a concentration-dependent reduction in lipid accumulation in *C. cohnii* [51]. Similarly, the fatty acid composition was altered in the presence of sesamol, whereby the stearic acid proportion of the TFA per cell dry weight decreased from 10% to 8.50% at 1.5 mM of sesamol, while the DHA proportion increased from 36.97% to 41.13% of the TFA at only 0.5 mM of sesamol [51]. Surprisingly, even though the weight content of all the fatty acids was reduced in the presence of sesamol, DHA was the least affected fatty acid [51]. The role that DHA plays in attenuating CVDs, hypertension, and diabetes disorders indicates that DHA is a potent polyunsaturated fatty acid with many health benefits [51]. Furthermore, DHA productivity was significantly elevated at 0.5–1 mM of sesamol but dropped when the concentration of sesamol reached 1.5 mM, which is an interesting finding that demands further notice [51]. Studies exploring the optimal dose of sesamol to be used in investigations found that sesamol doses lower than 300 mg/kg produced the most favorable effects [52]. Interestingly, doses between the ranges of 300 and 2000 mg/kg were found to be associated with significant toxicological effects, whereby administration of sesamol at a 2000 mg/kg dose to C57BL/6 mice was found to result in severe histopathological changes in all organs and excess DNA strand breakage [52]. While inconclusive, these findings suggest that future pre-clinical, clinical, and drug development investigations on sesamol should employ doses that do not exceed 300 mg/kg to observe maximal therapeutic effects. Similarly, an *in vivo* study by Bao and colleagues evaluated the molecular mechanisms underlying the potential of sesamol (0.5–7 mM) in regulating lipid synthesis and DHA synthesis in *Schizochytrium* sp. H016 [53]. Interestingly, lower concentrations of sesamol (up to 1.5 mM) were shown to exert a stimulatory effect on lipid accumulation and DHA synthesis, most likely owing to the intrinsic fatty acid synthesis pathways of *Schizochytrium* sp., while higher doses (5–7 mM) inhibited lipid synthesis [53]. These findings suggest that sesamol may have different effects on lipid synthesis depending on the oleaginous microorganism [53]. Additionally, similar to findings reported by Liu and colleagues, results from this study indicated a sesamol-mediated shift in TFA composition, whereby the synthesis of PUFAs such as DHA increased at the expense of saturated fatty acid synthesis [53]. Moreover, this study further confirms that sesamol decreases the nicotinamide adenine dinucleotide phosphate hydrogen (NADPH) supply by significantly reducing the activity of the malic enzyme, a key enzyme required for producing NADPH and catalyzing the reactions involved in lipogenesis [54]. In particular, a 22.81% reduction in NADPH content was observed in the presence of 1 mM of sesamol, resulting from the sesamol-mediated reduction in malic enzyme activity [53]. The malic enzyme is known to be dually regulated by both *PPARα* and *SREBP-1* [55], two pathways shown to be regulated in opposite directions by sesamol, with the former being increased and the latter decreased. If malic enzyme is regulated by these two opposing pathways solely, one would expect sesamol to only exert small to moderate effects on malic enzyme, which was not the case in the studies reported in this review, as malic enzyme was found to be significantly reduced by sesamol. It remains to be clarified the magnitude of the effect sesamol exerts on malic enzyme specifically and the effect of sesamol on the *PPARα* pathway in the context of fatty acid synthesis, as it was not explored in the context of fatty acid synthesis in this review. Perhaps more interestingly, results from this study and others may suggest that other signaling pathways may also be at play in regulating the expression of malic enzyme, a possibility that warrants further future research. The reported *in vivo* and *in vitro* effects of sesamol on fatty acid synthesis are summarized in Table 2.

### 4.2. Effects of Sesamol on Fatty Acid Oxidation

In addition to its effects on fatty acid synthesis, sesamol has also been shown to enhance both mitochondrial and hepatic peroxisomal fatty acid oxidation rates. Multiple studies point to the role that sesamol can play on *PPARα*, a key transcription factor that regulates gene expression in fatty acid β-oxidation [56]. For example, in a combined *in vivo* and *in vitro* study, the effects of sesamol on hepatic steatosis were evaluated in HFD-induced obese mice and palmitic acid (PA)-induced HepG2 cells [38]. Sesamol was found to elevate the levels of β-Hydroxybutyric acid (β-HB), a fatty acid metabolite that results from the fatty acid β-oxidation of free fatty acids (FFAs), which in turn had been reduced [38]. These changes were observed in the livers of the obese mice and HepG2 cells, and further molecular analysis revealed that sesamol had upregulated the levels of *PPARα*, peroxisome proliferator-activated receptor-gamma coactivator-1α (*PGC1α*), and carnitine palmitoyltransferase-1α (*CPT1α*) [38]. Noteworthy, *CPT1α* has been well established in the literature as the key rate-limiting enzyme in fatty acid oxidation [57]. In this study, sesamol was also found to activate the *AMPK* signaling pathway in both experimental models by elevating the *p-AMPK/AMPK* ratio, which most likely regulates hepatic lipogenesis and fatty acid β-oxidation [38]. The *AMPK* pathway stimulates a biochemical process called lipolysis that generates FFAs, which are eliminated by fatty acid oxidation. Similar results were reported by this *in vitro* investigation in which high oleic acid/cholesterol-induced HepG2 cells were treated with sesamol (0.75, 1.5, and 3 μg/mL) for 36 h to study the effects of sesamol on hepatic steatosis [39]. Notably, sesamol exerted promoting effects on fatty acid oxidation through the upregulation of *PPARα* gene expression in the hepatic tissue and the dose-dependent elevation of *CPT1A* gene expression [39]. It remains to be clarified, however, whether *CPT1a* and *PGC1α* are regulated by *PPARα* alone or by a combination of different signaling pathways. If the latter is true, research should certainly pinpoint the other potential pathways involved to gain a better understanding of the molecular pathways underlying fatty acid oxidation and where in the process sesamol can interfere. In another study, the administration of sesamol (0.05% *w/v*) to HFFD-fed C57BL/6J mice demonstrated significant *in vivo* effects on fatty acid oxidation, whereby sesamol was shown to upregulate the gene expression of *CPT1* and *CPT2* enzymes, which regulate the mitochondrial trifunctional protein (MTP) that carries out the mitochondrial β-oxidation of fatty acids [58]. In another *in vitro* study, Go and colleagues reported that sesamol exerted promoting effects on fatty acid oxidation in 3T3-L1 cells [48]. Precisely, sesamol (0, 50, 100, and 150 μM), which was administered to the 3T3-L1 cells for 24 h, was found to activate the *AMPK* signaling pathway, as evidenced by the inhibition of TG synthesis and the enhancement of fatty acid oxidation [48]. Sesamol was also shown to upregulate the levels of hormone-sensitive lipase (HSL) and lipoprotein lipase (LPL), which are involved in lipolysis [48]. In an *in vivo* study on the therapeutic effects of sesamol on obesity induced by HFD in mice, the ingestion of sesamol (100 mg/kg body weight/day) by gavage in male C57BL/6J mice for 4 weeks significantly promoted hepatic lipid metabolism [30]. Precisely, sesamol was found to enhance the function of hepatic lipid metabolism regulators, which are involved in the downregulation of SREBP-1c, a critical transcription factor for the induction of lipolysis, and the upregulation of phosphorylated HSL (*p-HSL*), *CPT1α*, and *PGC1α*, which enhanced lipolysis and fatty acid β-oxidation and reduced fatty acid synthesis [30]. Surprisingly, another study that observed the activity of SREBP1 reported very different findings. In this *in vitro* study that was conducted on human mesenchymal stem cells (hMSCs), Kim and colleagues assessed the effects of sesamol (1, 10, 50, and 100 mM) treatment in hMSCs for 14 days on markers of fatty acid oxidation [59]. Remarkably, sesamol was not shown to exert any effects on lipolysis [59]. Further molecular investigation revealed that the absence of a sesamol-mediated effect on lipolysis was due to the inability of sesamol to downregulate the expression of *SREBP1* [59]. Overall, the role that sesamol plays in upregulating fatty acid oxidation is promising. Future *in vivo* and *in vitro* investigations may benefit from examining the effects of sesamol on early- and late-stage β-oxidation enzymes to better understand which genes, proteins, and pathways are specifically targeted. The reported *in vitro* and *in vivo* effects of sesamol on fatty acid oxidation are summarized in Table 3.

### 4.3. Effects of Sesamol on Cholesterol Metabolism

Cholesterol plays a critical role in many metabolic processes that are necessary for normal cell functioning [60]. Despite its critical role, keeping serum cholesterol levels under control is imperative, as an increase in their levels can result in atherosclerosis, resulting in plaque buildup and reduced blood flow [61]. In this regard, a number of studies have reported findings that demonstrate the role that sesamol may play in keeping cholesterol within suitable levels. In the context of cholesterol synthesis, sesamol has been shown to reduce the activity of 3-hydroxy-3-methylglutaryl-CoA reductase (HMGCR), a key enzyme that catalyzes the rate-limiting reaction in cholesterol biosynthesis [60]. For instance, in an *in vivo* study on HFFD-induced C57BL/6J mice, the oral administration of sesamol (0.05% *w/v*) to the mice revealed significant effects on cholesterol metabolism [58]. In particular, sesamol was shown to downregulate the gene expression of HMGCR and acetyl-CoA acetyltransferase 2 (ACAT2), both of which have been shown to be essential in the cholesterol synthesis pathway [58]. Additionally, results from this study revealed that sesamol remarkably improved the gene expression of *LXRα* and *LXRβ*, nuclear receptors that enhance cholesterol excretion, and ATP-binding cassette sub-family G members 5 (*ABCG5*) and ATP-binding cassette sub-family G member 5 (*ABCG8*), which facilitate the secretion of cholesterol in the form of bile acids [58].

In addition to its reported inhibitory effects on HMGCR activity, sesamol was also shown to promote cholesterol homeostasis by acting on several genes and proteins known to play critical roles in cellular cholesterol and phospholipid regulation. For example, in an *in vitro* study, sesamol was found to upregulate the expression levels of *PPARs* downstream genes in high oleic acid/cholesterol-induced HepG2 cells [39]. Precisely, sesamol (0.75, 1.5, and 3 μg/mL) treatment for 36 h was shown to upregulate the expression levels of the *PPARs* downstream genes *LXRα*, ATP-binding cassette subfamily A member 1 (*ABCA1*), and cytochrome P450 family 7 subfamily A member 1 (*CYP7A1*), which encode an enzyme that catalyzes the first step in cholesterol catabolism and bile acid synthesis, which in turn concurrently enhanced cholesterol metabolism and lowered serum LDL-C levels [39,62]. These findings were further corroborated in another *in vivo* study, whereby sesamol was found to exert significant effects on serum lipid profiles via exerting inhibitory effects on cholesterol biosynthesis in DXO-induced Wistar rats [37]. Specifically, sesamol (50 mg/kg of body weight) pretreatment for 7 days followed by DXO administration for 2 weeks was found to inhibit cholesterol biosynthesis and enhance LDL-C uptake from the bloodstream by the liver [37]. Although promising, research exploring the possible effects of sesamol on other aspects of cholesterol metabolism, such as absorption, excretion, and the levels of free cholesterol in serum and blood, is largely lacking. Studies looking at these aspects are certainly warranted to further substantiate the potential of a natural lignan such as sesamol in regulating cholesterol homeostasis. Additionally, studies comparing the use of sesamol to that of statins for cholesterol regulation may be useful to help investigate the potential of using such a lignan clinically against hypercholesterolemia. The reported *in vivo* and *in vitro* effects of sesamol on cholesterol metabolism are summarized in Table 4.

### 4.4. Macrophage Cholesterol Efflux

Aside from its effects on cholesterol metabolism, sesamol could also potentially have an effect on promoting macrophage cholesterol homeostasis. Studies have confirmed that the accumulation of cholesterol in macrophages in the artery walls causes an immune response that may initiate atherosclerosis; therefore, the efflux of cholesterol from macrophages is a crucial step in preventing atherogenesis. In one *in vitro* study, sesamol (25, 50, 75, and 100 μM) and sesame oil (1, 2.5, 5, and 10 μg/mL) were shown to ameliorate macrophage cholesterol efflux via upregulating the expression of the nuclear receptors peroxisome proliferator-activated receptor γ 1 (*PPARγ1*) and *LXRα* in Chinese hamster ovary (CHO) cells [63]. In particular, the treatment with 75 and 100 μM of sesamol caused a significant 1.7-fold increase in both *PPARγ1* and *LXRα* expressions [63]. Additionally, further molecular analysis revealed that adding 100 μM of sesamol caused a fourfold elevation of the *PPARγ1* transcriptional activity as well as a 2.2-fold elevation of the LXRα transcriptional activity [63]. Previous studies have shown that the mitogen-activated protein kinase (*MAPK*) signaling pathway is essential for cholesterol homeostasis and modified LDL uptake by macrophages [63]. This study reported that the *MAPK* signaling pathway is also heavily involved in the sesamol-mediated upregulation of *PPARγ1* and *LXRα* expression [63]. Furthermore, after treating the macrophages with apolipoprotein AI (ApoAI) to induce cholesterol efflux, 100 μM sesamol in particular was found to cause a 2.6-fold enhancement of ApoAI-specific macrophage cholesterol efflux [63]. Although compelling, as it stands, evidence confirming the role sesamol may play in promoting cholesterol homeostasis via enhancing macrophage cholesterol efflux is largely lacking. Specifically, future studies exploring the possible direct effects of sesamol on foam cell accumulation and on other genes that are known to be heavily involved in coding for proteins that promote cholesterol efflux, such as *ABCA1* and *ABCG1,* are certainly warranted. If confirmed, this can hold great potential for the use of sesamol as a natural hypocholesterolemic agent with minimal side effects. The reported *in vitro* effects of sesamol on macrophage cholesterol efflux are summarized in Table 5.

## 5. Clinical Significance and Design of Preclinical, Clinical, and Preventive Studies on the Hypolipidemic Effects of Sesamol

As outlined above, several *in vitro* and *in vivo* experimental findings suggest that sesamol may be of clinical significance as it can potentially be implicated as a hypolipidemic agent for the regulation of serum lipid levels, fatty acid synthesis and oxidation, cholesterol metabolism, and macrophage cholesterol efflux. Although the preclinical and experimental evidence suggesting potent hypolipidemic effects of sesamol is compelling, preventive, clinical studies that directly point to the hypolipidemic and anti-atherogenic potential of sesamol in patients with different types of dyslipidemia are still lacking. Several *in vivo* studies using different models of mice and rats were conducted to assess the hypolipidemic potential of sesamol. However, no single human study was conducted to evaluate the clinical efficacy of sesamol as a possible lipid-lowering, anti-atherogenic agent. Based on the compelling *in vitro* and *in vivo* experimental evidence suggesting that sesamol may be an effective hypolipidemic agent, we herein propose well-designed, randomized, placebo-controlled clinical studies. In one investigation, age-matched patients with the same level of atherogenic dyslipidemia could be divided into three groups. Group I patients will receive statins (10 mg, twice daily), group II patients will receive statins (10 mg, twice daily) plus sesamol (10 mg, twice daily), group III patients will receive statins (10 mg, twice daily) plus sesamol (50 mg, twice daily), and group IV patients will receive statins (10 mg, twice daily) plus sesamol (100 mg, twice daily) for 6 months. Based on studies performed on mice and rats, these doses are subtoxic and cause no biosafety concerns. The prognosis and clinical parameters of atherogenic dyslipidemia in all patients are to be evaluated during and after the clinical trial. We also propose a longitudinal hyperlipidemia prevention study to evaluate the potential preventive effects of sesamol against atherosclerosis. To this end, age- and gender-matched healthy individuals (age > 60 years) can be divided into three groups. Group I participants will receive a placebo (control), group II participants will receive sesamol (10 mg, twice daily), group III participants will receive sesamol (50 mg, twice daily), and group IV participants will receive sesamol (100 mg, twice daily) for 2–3 years. All participants will be closely and systematically monitored for the emergence of atherogenic dyslipidemia. These studies could be very helpful in establishing links between the reported regulatory effects of sesamol on serum lipid profile, fatty acid synthesis and oxidation, cholesterol metabolism, and macrophage cholesterol efflux levels and atherosclerosis prevention and/or treatment in preclinical and clinical settings.

## 6. Conclusions

**Figure 1:**
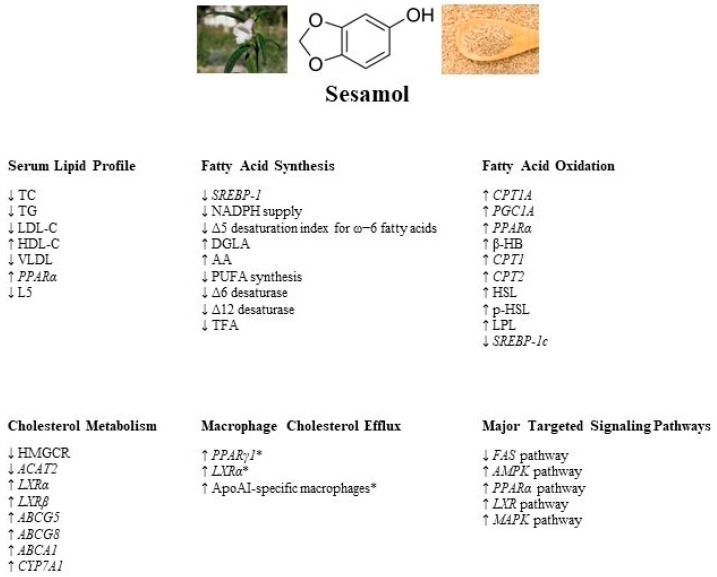
A summary of the regulatory effects of sesamol on serum lipid profile and signaling factors and proteins involved in fatty acid synthesis, fatty acid oxidation, cholesterol metabolism, and macrophage cholesterol homeostasis. The major signaling pathways targeted by sesamol are denoted as well. (* with the addition of sesame oil). TC: total cholesterol; TG: triglyceride; LDL-C: low-density lipoprotein cholesterol; HDL: high-density lipoprotein cholesterol; VLDL: very low-density lipoprotein; PPARα: peroxisome proliferator-activated receptor α; SREBP-1: sterol regulatory element binding protein-1; NADPH: nicotinamide adenine dinucleotide phosphate; DGLA: dihomo-γ-linolenic acid; AA: arachidonic acid; PUFA: polyunsaturated fatty acid; TFA: total fatty acids; CPT1A: carnitine palmitoyltransferase-1A; PGC1A: peroxisome proliferator-activated receptor-gamma coactivator-1A; β-HB: β-hydroxybutyric acid; HSL: hormone-sensitive lipase; pHSL: phosphorylated hormone-sensitive lipase; LPL: lipoprotein lipase; HMGCR: 3-hydroxy-3-methylglutaryl-CoA reductase; ACAT2: acetyl-CoA acetyltransferase 2; LXRα: liver X receptor α; LXRβ: liver X receptor α; ABCG5: ATP-binding cassette sub-family G member 5; ABCG8: ATP-binding cassette sub-family G member 8; ABCA1: ATP-binding cassette sub-family A member 1; CYP7A1: cytochrome P450 family 7 subfamily A member 1; PPARγ1: peroxisome proliferator-activated receptor γ 1; ApoAI: apolipoprotein AI; FAS: fatty acid synthase; AMPK: AMP-activated protein kinase; MAPK: mitogen-activated protein kinase.

The present comprehensive review indicates that sesamol, a lignan found in *S. indicum* seeds, exerts potent lipid-lowering effects in various *in vivo* and *in vitro* models and could potentially be employed as an anti-atherogenic agent. Sesamol is believed to exert its lipid-lowering effects via reduction in fatty acid synthesis, induction of fatty acid oxidation, enhancement of cholesterol metabolism, and modulation of macrophage cholesterol efflux. Figure 1 provides a summary of the regulatory effects of sesamol on the signaling factors and proteins involved in the molecular mechanisms underlying the sesamol-mediated reduction in lipid levels. Sesamol reduces fatty acid synthesis mainly by downregulating the *SREBP-1c*, *FAS*, Δ6, and Δ12 desaturase enzyme expression and activating the *AMPK* signaling pathway. Noteworthy, the reported literature does not show that sesamol reduces the activity of Δ5 desaturase, which is linked to fatty acid synthesis. Moreover, sesamol induces fatty acid β-oxidation and concomitantly reduces serum FFA levels via upregulating *PPARα*, *PGC1α*, *CPT1A*, and *CPT2* gene expression. A few studies reported that sesamol enhances cholesterol metabolism by downregulating HMGCR, a key enzyme in cholesterol synthesis, and upregulating the *LXRα* regulatory pathway, involved in macrophage cholesterol efflux. More studies are certainly warranted to examine the underlying mechanisms by which sesamol improves cholesterol metabolism and maintains macrophage cholesterol efflux. It is important to note that most of the *in vivo* studies reported in this review used rodent models, including mice, rats, and hamsters. Given the species-specific discrepancies in lipid metabolism between humans and rodents, replicating the reported findings in humans is imperative in order to consider sesamol as a therapeutic lipid-lowering, anti-atherogenic agent. Nonetheless, as it stands, sesamol appears to possess potent anti-hyperlipidemic and anti-atherogenic properties, making it a possible novel treatment for hyperlipidemia and related CVDs with minimal side effects.

## Figures and Tables

**Table 1:** The effects of sesamol and its derivatives on serum lipid profile.

Main Effect	Experimental Model	Dosage	Administration Mode	Administration Duration	Ref.
Reduction of TC, TG, and LDL-C levels Elevation of HDL-C levels Upregulation of hepatic *PPARα* expression Downregulation of *LXRα*, *SREBP-1c*, FAS expression Reduction of fatty acid synthesis	Male Wistar albino rats	2, 4, and 8 mg/kg/day	Oral gavage	Treatment for 30 days	[29]
Reduction of serum TC and LDL-C levels Elevation of HDL-C levels	Male C57BL/6J mice	100 mg/kg body weight/day	Gavage	Treatment for 4 weeks	[30]
Suppression of lipid accumulation Reduction of TC and TG levels	Male C57BL/6J mice	100 mg/kg body weight/day	Gavage	Treatment for 8 weeks	[31]
Reduction of TG, TC, and LDL-C Elevation of HDL-C	Male Wistar rats	10 and 20 mg/kg	Oral administration	Pretreatment for 60 days (45 min prior to administering AlCl_3_)	[32]
Reduction of elevated TG levels Normalizing TC level Counteraction of tyloxapol-induced elevation of plasma cholesterol	Male Swiss Albino mice	100 and 200 mg/kg (Acute model) 200 mg/kg (Tyloxapol-induced mice) 50 and 100 mg/kg (HFFD-induced mice)	Oral administration	Treatment for 240 min (Acute model) Treatment for 24 h (Tyloxapol-induced mice) Treatment for 45 days (HFD-induced mice)	[33]
Reduction of LDL-C, VLDL, and TC serum levels Reduction of L5 serum level Reduction of atherosclerotic lesion size	Male Syrian hamsters	50 or 100 mg/kg	Oral gavage	Treatment for 16 weeks	[34]
Reduction of plasma TC, LDL-C, VLDL, and TG levels Elevation of plasma HDL-C levels	Male Albino Wistar rats	50, 100, and 200 mg/kg of body weight	Intraperitoneal administration	Treatment for 7 days	[36]
Reduction of serum TC, TG, LDL-C, and VLDL levels Elevation of serum HDL-C level Inhibition of myocardial necrosis	Albino Wistar rats	50 mg/kg of body weight	Not available	Pretreatment for 7 days	[37]
Reduction of hepatic fat vacuoles and liver weight Reduction of serum ALT and AST levels Reduction of serum TG and LDL-C levels	Male C57BL/6J mice	100 mg/kg body weight/day	Gavage	Treatment for 8 weeks	[38]
Reduction of intracellular TG and TC levels Activation of *PPAR* signaling pathway	HepG2 cells	0.75, 1.5, and 3 μg/mL	Not available	Treatment for 36 h	[39]
Reduction of serum TG, TC, and LDL-C levels Activation of *PPAR*α	Hyperlipidemic mice	N/A (sesamol-based fibrate derivative compound **12**)	Not available	Not available	[40]
Reduction of plasma TG and TC levels Reduction of LDL-C levels Activation of *PPAR*α receptor Suppression of lipid accumulation	KM mice	0.36 mmol/kg (compounds **T1**–**T15**)	Intragastric administration	Treatment for 7 days	[41]
	KM mice	0.36 mmol/kg (compound **T6**)	Intragastric administration	Treatment for 4 weeks	
Reduction of plasma TG and TC levels	KM mice	0.36 mmol/kg (CF-sesamol)	Intragastric administration	Treatment for 1 month	[42]

**Table 2:** The effects of sesamol on fatty acid synthesis.

Main Effect	Experimental Model	Dosage	Administration Mode	Administration Duration	Ref.
Reduction of *FAS* and *SREBP-1* expression levels Activation of *AMPK* signaling pathway	3T3-L1 cells	0, 50, 100, and 150 μM	Not available	Treatment for 24 h	[48]
Downregulation of *SREBP-1c* and *FAS* expression Reduction of fatty acid synthesis	Male Wistar albino rats	2, 4, and 8 mg/kg/day	Oral gavage	Treatment for 30 days	[29]
Reduction of LDs	HepG2 cells	0.75, 1.5, and 3 μg/mL	Not available	Treatment for 36 h	[39]
Reduction of the Δ5 desaturation index for ω−6 fatty acids Elevation of DGLA levels Reduction of AA levels Reduction of PUFA synthesis Reduction of DPA levels No effect on the levels of saturated and monounsaturated fatty acids and linoleic acid	Female BALB/c mice	1 wt%	Oral administration	Treatment for 14 days	[49]
Reduction of the activities of Δ6 and Δ12 desaturases Elevation of oleic acid and stearic acid levels Reduction of pyruvate carboxylase and NADP+/isocitrate dehydrogenase activities Reduction of malic enzyme and fatty acid synthetase activities Reduction of NADPH supply	*Mucor circinelloides*	0–10.1 mM	Not available	Not available	[50]
Reduction of TFA content and intracellular ROS levels Reduction of stearic acid percentage of TFA and lipid accumulation Elevation of DHA % of TFA and DHA productivity	*Crypthecodinium cohniiz*	0.5, 1, 1.5, and 2 mM	Not available	Not available	[51]
Elevation of lipid accumulation and DHA synthesis Elevation of PUFA synthesis Reduction of saturated fatty acid synthesis Reduction of malic enzyme activity and NADPH supply	*Schizochytrium* sp. H016	0.5–7 mM	Not available	Not available	[52]

**Table 3:** The effects of sesamol on fatty acid oxidation.

Main Effect	Experimental Model	Dosage	Administration Mode	Administration Duration	Ref.
Activation of *AMPK* signaling pathway Inhibition of TG synthesis Enhancement of fatty acid oxidation Upregulation of HSL and LPL levels	3T3-L1 cells	0, 50, 100, and 150 μM	Not available	Treatment for 24 h	[48]
Elevation of β-HB level Elevation of fatty acid β-oxidation Reduction of serum FFA levels Upregulation of *PPARα*, *PGC1α*, and *CPT1α* levels Activation of *AMPK* signaling pathway Elevation of *p-AMPK/AMPK* ratio	Male C57BL/6J mice	100 mg/kg body weight/day (C57BL/6J mice)	Gavage (C57BL/6J mice)	Treatment for 8 weeks (C57BL/6J mice)	[38]
	HepG2 cells	12.5, 25, and 50 µM	N/A (HepG2 cells)	Treatment for 24 h (HepG2 cells)	
Upregulation of hepatic *PPARα* gene expression Elevation of *CPT1A* gene expression	HepG2 cells	0.75, 1.5, and 3 μg/mL	Not available	Treatment for 36 h	[39]
Upregulation of *CPT1* and *CPT2* gene expression	C57BL/6J mice	0.05% *w/v*	Oral administration	Treatment for 12 weeks	[58]
Downregulation of *SREBP-1c* Upregulation of p-*HSL*, *CPT1α*, and *PGC1α* Enhancement of lipolysis and fatty acid β-oxidation	Male C57BL/6J mice	100 mg/kg body weight/day	Gavage	Treatment for 4 weeks	[30]
No effect on lipolysis No effect on *SREBP1* expression	hMSCs	1, 10, 50, and 100 mM	Not available	Treatment for 14 days	[59]

**Table 4:** The effects of sesamol on cholesterol metabolism.

Main Effect	Experimental Model	Dosage	Administration Mode	Administration Duration	Ref.
Downregulation of HMGCR and *ACAT2* gene expression Improvement *of LXRα*, *LXRβ*, *ABCG5*, and *ABCG8* gene expression	C57BL/6J mice	0.05% *w*/*v*	Oral administration	Treatment for 12 weeks	[58]
Upregulation of *PPARs* downstream genes (*LXRα*, *ABCA1*, and *CYP7A1*) Enhancement of cholesterol metabolism Reduction of serum LDL-C levels	HepG2 cells	0.75, 1.5, and 3 μg/mL	Not available	Treatment for 36 h	[39]

**Table 5:** The effects of sesamol on macrophage cholesterol efflux.

Main Effect	Experimental Model	Dosage	Administration Mode	Administration Duration	Ref.
Upregulation of *PPARγ1* and *LXRα* expression via *MAPK* pathway Elevation of *PPARγ1* and *LXRα* transcriptional activity Enhancement of ApoAI-induced macrophage cholesterol efflux	CHO cells	25, 50, 75, and 100 μM	Not available	Treatment for 24 h	[63]

## Data Availability

Not applicable.

## References

[1] Alloubani A., Nimer R., Samara R. (2021). Relationship between Hyperlipidemia, Cardiovascular Disease and Stroke: A Systematic Review. Curr. Cardiol. Rev..

[2] Li Z.-Z., Huang Q., Yang X., Zeng J., Wang Q.-H., Tang H.-M., Yu Z., Song Y.-Q., Liu Y. (2022). Cholesterol Metabolic Markers for Differential Evaluation of Patients with Hyperlipidemia and Familial Hypercholesterolemia. Dis. Markers.

[3] Vekic J., Zeljkovic A., Cicero A.F.G., Janez A., Stoian A.P., Sonmez A., Rizzo M. (2022). Atherosclerosis Development and Progression: The Role of Atherogenic Small, Dense LDL. Medicina.

[4] Thompson P.D., Panza G., Zaleski A., Taylor B. (2016). Statin-Associated Side Effects. J. Am. Coll. Cardiol..

[5] Sattar N., Preiss D., Murray H.M., Welsh P., Buckley B.M., de Craen A.J., Seshasai S.R., McMurray J.J., Freeman D.J., Jukema J.W. (2010). Statins and Risk of Incident Diabetes: A Collaborative Meta-Analysis of Randomised Statin Trials. Lancet.

[6] Desam N.R., Al-Rajab A.J. (2022). Herbal Biomolecules: Anticancer Agents. Herbal Biomolecules in Healthcare Application.

[7] Parikh M., Netticadan T., Pierce G.N. (2018). Flaxseed: Its Bioactive Components and Their Cardiovascular Benefits. Am. J. Physiol.-Heart Circ..

[8] Anilakumar K.R., Pal A., Khanum F., Bawa A.S. (2010). Nutritional, Medicinal and Industrial Uses of Sesame (*Sesamum indicum* L.) seeds-an overview. Agric. Conspec. Sci..

[9] Wang J.S., Tsai P.H., Tseng K.F., Chen F.Y., Yang W.C., Shen M.Y. (2021). Sesamol Ameliorates Renal Injury-Mediated Atherosclerosis via Inhibition of Oxidative Stress/IKKα/p53. Antioxidants.

[10] Andargie M., Vinas M., Rathgeb A., Möller E., Karlovsky P. (2021). Lignans of Sesame (*Sesamum Indicum* L.): A Comprehensive Review. Molecules.

[11] Wu M.S., Aquino L.B., Barbaza M.Y., Hsieh C.L., Castro-Cruz K.A., Yang L.L., Tsai P.W. (2019). Anti-Inflammatory and Anticancer Properties of Bioactive Compounds from *Sesamum indicum* L.-A Review. Molecules.

[12] Wei P., Zhao F., Wang Z., Wang Q., Chai X., Hou G., Meng Q. (2022). Sesame (*Sesamum indicum* L.): A Comprehensive Review of Nutritional Value, Phytochemical Composition, Health Benefits, Development of Food, and Industrial Applications. Nutrients.

[13] Siriwarin B., Weerapreeyakul N. (2016). Sesamol Induced Apoptotic Effect in Lung Adenocarcinoma Cells through both Intrinsic and Extrinsic Pathways. Chem. Biol. Interact..

[14] Srisayam M., Weerapreeyakul N., Barusrux S., Kanokmedhakul K. (2014). Antioxidant, Antimelanogenic, and Skin-Protective Effect of Sesamol. J. Cosmet. Sci..

[15] Liou C.-J., Chen Y.-L., Yu M.-C., Yeh K.-W., Shen S.-C., Huang W.-C. (2020). Sesamol Alleviates Airway Hyperresponsiveness and Oxidative Stress in Asthmatic Mice. Antioxidants.

[16] Bosebabu B., Cheruku S.P., Chamallamudi M.R., Nampoothiri M., Shenoy R.R., Nandakumar K., Parihar V.K., Kumar N. (2020). An Appraisal of Current Pharmacological Perspectives of Sesamol: A Review. Mini Rev. Med. Chem..

[17] Zheng W., Song Z., Li S., Hu M., Shaukat H., Qin H. (2021). Protective Effects of Sesamol against Liver Oxidative Stress and Inflammation in High-Fat Diet-Induced Hepatic Steatosis. Nutrients.

[18] Kondamudi P.K., Kovelamudi H., Mathew G., Nayak P.G., Rao C.M., Shenoy R.R. (2013). Modulatory Effects of Sesamol in Dinitrochlorobenzene-Induced Inflammatory Bowel Disorder in Albino Rats. Pharmacol. Rep..

[19] Narasimhulu C.A., Selvarajan K., Litvinov D., Parthasarathy S. (2015). Anti-Atherosclerotic and Anti-Inflammatory Actions of Sesame Oil. J. Med. Food..

[20] Kapadia G.J., Azuine M.A., Tokuda H., Takasaki M., Mukainaka T., Konoshima T., Nishino H. (2002). Chemopreventive Effect of Resveratrol, Sesamol, Sesame Oil and Sunflower Oil in the Epstein-Barr Virus Early Antigen Activation Assay and the Mouse Skin Two-Stage Carcinogenesis. Pharmacol. Res..

[21] Majdalawieh A.F., Mansour Z.R. (2019). Sesamol, a Major Lignan in Sesame Seeds (*Sesamum indicum*): Anti-Cancer Properties and Mechanisms of Action. Eur. J. Pharmacol..

[22] Jayaraj P., Narasimhulu C.A., Rajagopalan S., Parthasarathy S., Desikan R. (2020). Sesamol: A Powerful Functional Food Ingredient from Sesame Oil for Cardioprotection. Food Funct..

[23] Tsai P.H., Chen L.Z., Tseng K.F., Chen F.Y., Shen M.Y. (2022). Apolipoprotein C3-Rich Low-Density Lipoprotein Induces Endothelial Cell Senescence via FBXO31 and Its Inhibition by Sesamol *In Vitro* and *In Vivo*. Biomedicines.

[24] Ying Z., Kherada N., Kampfrath T., Mihai G., Simonetti O., Desikan R., Selvendiran K., Sun Q., Ziouzenkova O., Parthasarathy S. (2011). A Modified Sesamol Derivative Inhibits Progression of Atherosclerosis. Arterioscler. Thromb. Vasc. Biol..

[25] Yang Y., Qu Y., Lv X., Zhao R., Yu J., Hu S., Kang J., Zhang Y., Gong Y., Cui T. (2021). Sesamol Supplementation Alleviates Nonalcoholic Steatohepatitis and Atherosclerosis in High-Fat, High Carbohydrate and High-Cholesterol Diet-Fed Rats. Food Funct..

[26] Karr S. (2017). Epidemiology and Management of Hyperlipidemia. Am. J. Manag. Care.

[27] Nelson R.H. (2013). Hyperlipidemia as a Risk Factor for Cardiovascular Disease. Prim. Care-Clin. Off. Pract..

[28] Cooney M.T., Dudina A., De Bacquer D., Wilhelmsen L., Sans S., Menotti A., De Backer G., Jousilahti P., Keil U., Thomsen T. (2009). HDL Cholesterol Protects against Cardiovascular Disease in Both Genders, at All Ages and at All Levels of Risk. Atherosclerosis.

[29] Sharma A.K., Bharti S., Bhatia J., Nepal S., Malik S., Ray R., Kumari S., Arya D.S. (2012). Sesamol Alleviates Diet-Induced Cardiometabolic Syndrome in Rats via Up-Regulating PPARγ, PPARα and E-NOS. J. Nutr. Biochem..

[30] Qin H., Xu H., Yu L., Yang L., Lin C., Chen J. (2019). Sesamol Intervention Ameliorates Obesity-Associated Metabolic Disorders by Regulating Hepatic Lipid Metabolism in High-Fat Diet-Induced Obese Mice. Food Nutr. Res..

[31] Lin C., Chen J., Hu M., Zheng W., Song Z., Qin H. (2021). Sesamol Promotes Browning of White Adipocytes to Ameliorate Obesity by Inducing Mitochondrial Biogenesis and Inhibition Mitophagy via β3-AR/PKA Signaling Pathway. Food Nutr. Res..

[32] Chamallamudi M., John J., Nampoothiri M., Kumar N., Mudgal J., Nampurath G. (2015). Sesamol, a Lipid Lowering Agent, Ameliorates Aluminium Chloride Induced Behavioral and Biochemical Alterations in Rats. Pharmacogn. Mag..

[33] Kumar N., Mudgal J., Parihar V.K., Nayak P.G., Kutty N.G., Rao C.M. (2013). Sesamol Treatment Reduces Plasma Cholesterol and Triacylglycerol Levels in Mouse Models of Acute and Chronic Hyperlipidemia. Lipids.

[34] Chen W.-Y., Chen F.-Y., Lee A.-S., Ting K.-H., Chang C.-M., Hsu J.-F., Lee W.-S., Sheu J.-R., Chen C.-H., Shen M.-Y. (2015). Sesamol Reduces the Atherogenicity of Electronegative L5 LDL *In Vivo* and *In Vitro*. J. Nat. Prod..

[35] Choi S.H., Ginsberg H.N. (2011). Increased Very Low Density Lipoprotein (VLDL) Secretion, Hepatic Steatosis, and Insulin Resistance. Trends Endocrinol. Metab..

[36] Vennila L., Pugalendi K.V. (2012). Efficacy of Sesamol on Plasma and Tissue Lipids in Isoproterenol-Induced Cardiotoxicity in Wistar Rats. Arch. Pharmacal Res..

[37] Chennuru A., Saleem M.T.S. (2013). Antioxidant, Lipid Lowering, and Membrane Stabilization Effect of Sesamol against Doxorubicin-Induced Cardiomyopathy in Experimental Rats. BioMed Res. Int..

[38] Xu H.-Y., Yu L., Chen J.-H., Yang L.-N., Lin C., Shi X.-Q., Qin H. (2020). Sesamol Alleviates Obesity-Related Hepatic Steatosis via Activating Hepatic PKA Pathway. Nutrients.

[39] Shi L., Karrar E., Wang X. (2021). Sesamol Ameliorates Hepatic Lipid Accumulation and Oxidative Stress in Steatosis HepG2 Cells via the PPAR Signaling Pathway. J. Food Biochem..

[40] Xie Y.-D., Xu Y.-H., Liu J.-P., Wang B., Shi Y.-H., Wang W., Wang X.-P., Sun M., Xu X.-Y., Bian X.-L. (2021). 1,3-Benzodioxole-Based Fibrate Derivatives as Potential Hypolipidemic and Hepatoprotective Agents. Bioorg. Med. Chem. Lett..

[41] Xie Y., Liu J., Shi Y., Wang B., Wang X., Wang W., Sun M., Xu X., He S. (2021). Synthesis and Evaluation of New Sesamol-Based Phenolic Acid Derivatives with Hypolipidemic, Antioxidant, and Hepatoprotective Effects. Med. Chem. Res..

[42] Xie Y., Liu J., Shi Y., Wang B., Wang X., Wang W., Sun M., Xu X., Jiang H., Guo M. (2021). The Combination of Sesamol and Clofibric Acid Moieties Leads to a Novel Potent Hypolipidemic Agent with Antioxidant, Anti-Inflammatory and Hepatoprotective Activity. Bioorg. Med. Chem. Lett..

[43] Majdalawieh A.F., Dalibalta S., Yousef S.M. (2020). Effects of Sesamin on Fatty Acid and Cholesterol Metabolism, Macrophage Cholesterol Homeostasis and Serum Lipid Profile: A Comprehensive Review. Eur. J. Pharmacol..

[44] Geerts H. (2009). Of Mice and Men. CNS Drugs.

[45] Lai M., Chandrasekera P.C., Barnard N.D. (2014). You Are What You Eat, or Are You? The Challenges of Translating High-Fat-Fed Rodents to Human Obesity and Diabetes. Nutr. Diabetes.

[46] Calder P.C. (2015). Functional Roles of Fatty Acids and Their Effects on Human Health. JPEN J. Parenter. Enter. Nutr..

[47] Siri-Tarino P.W., Sun Q., Hu F.B., Krauss R.M. (2010). Saturated Fat, Carbohydrate, and Cardiovascular Disease. Am. J. Clin. Nutr..

[48] Go G., Sung J.-S., Jee S.-C., Kim M., Jang W.-H., Kang K.-Y., Kim D.-Y., Lee S., Shin H.-S. (2017). *In Vitro* Anti-Obesity Effects of Sesamol Mediated by Adenosine Monophosphate-Activated Protein Kinase and Mitogen-Activated Protein Kinase Signaling in 3T3-L1 Cells. Food Sci. Biotechnol..

[49] Chavali S.R., Forse R.A. (1999). Decreased Production of Interleukin-6 and Prostaglandin E2 Associated with Inhibition of Delta5 Desaturation of ω6 Fatty Acids in Mice Fed Safflower Oil Diets Supplemented with Sesamol. Prostaglandins Leukot. Essent. Fat. Acids.

[50] Wynn J.P., Kendrick A., Ratledge C. (1997). Sesamol as an Inhibitor of Growth and Lipid Metabolism in Mucor Circinelloides via Its Action on Malic Enzyme. Lipids.

[51] Liu B., Liu J., Sun P., Ma X., Jiang Y., Chen F. (2015). Sesamol Enhances Cell Growth and the Biosynthesis and Accumulation of Docosahexaenoic Acid in the Microalga *Crypthecodinium Cohnii*. J. Agric. Food Chem..

[52] Khan S., Choudhary S., Kumar A., Tripathi A.M., Alok A., Adhikari J.S., Rizvi M.A., Chaudhury N.K. (2016). Evaluation of Sesamol-Induced Histopathological, Biochemical, Haematological and Genomic Alteration after Acute Oral Toxicity in Female C57bl/6 Mice. Toxicol. Rep..

[53] Bao Z., Zhu Y., Feng Y., Zhang K., Zhang M., Wang Z., Yu L. (2022). Enhancement of Lipid Accumulation and Docosahexaenoic Acid Synthesis in Schizochytrium Sp. H016 by Exogenous Supplementation of Sesamol. Bioresour. Technol..

[54] Jiang P., Du W., Mancuso A., Wellen K.E., Yang X. (2013). Reciprocal Regulation of p53 and Malic Enzymes Modulates Metabolism and Senescence. Nature.

[55] Ide T., Azechi A., Kitade S., Kunimatsu Y., Suzuki N., Nakajima C. (2012). Combined Effect of Sesamin and α-Lipoic Acid on Hepatic Fatty Acid Metabolism in Rats. Eur. J. Nutr..

[56] van Raalte D.H., Li M., Pritchard P.H., Wasan K.M. (2004). Peroxisome Proliferator-Activated Receptor (PPAR)-Alpha: A Pharmacological Target with a Promising Future. Pharm. Res..

[57] Schlaepfer I.R., Joshi M. (2020). CPT1A-Mediated Fat Oxidation, Mechanisms, and Therapeutic Potential. Endocrinology.

[58] Liu Z., Qiao Q., Sun Y., Chen Y., Ren B., Liu X. (2017). Sesamol Ameliorates Diet-Induced Obesity in C57BL/6J Mice and Suppresses Adipogenesis in 3T3-L1 Cells via Regulating Mitochondria-Lipid Metabolism. Mol. Nutr. Food Res..

[59] Kim M., Lee Y.-J., Jee S.-C., Choi I., Sung J.-S. (2016). Anti-Adipogenic Effects of Sesamol on Human Mesenchymal Stem Cells. Biochem. Biophys. Res. Commun..

[60] Cortes V.A., Busso D., Maiz A., Arteaga A., Nervi F., Rigotti A. (2014). Physiological and Pathological Implications of Cholesterol. Front. Biosci..

[61] Ruotsalainen A.-K., Mäkinen P., Ylä-Herttuala S. (2021). Novel RNAi-Based Therapies for Atherosclerosis. Curr. Atheroscler. Rep..

[62] Pullinger C.R., Eng C., Salen G., Shefer S., Batta A.K., Erickson S.K., Verhagen A., Rivera C.R., Mulvihill S.J., Malloy M.J. (2002). Human Cholesterol 7α-Hydroxylase (CYP7A1) Deficiency Has a Hypercholesterolemic Phenotype. J. Clin. Investig..

[63] Majdalawieh A.F., Ro H.-S. (2014). Sesamol and Sesame (*Sesamum indicum*) Oil Enhance Macrophage Cholesterol Efflux via Up-Regulation of PPARγ1 and LXRα Transcriptional Activity in a MAPK-Dependent Manner. Eur. J. Nutr..

